# Sodium, potassium intake and urinary sodium-to-potassium ratio in rheumatoid arthritis: association with markers of cardiovascular dysfunction and disease-related parameters

**DOI:** 10.1007/s10067-025-07608-4

**Published:** 2025-08-01

**Authors:** Panagiota Anyfanti, Christina Antza, Alexandra Ainatzoglou, Elena Angeloudi, Smaro Palaska, Dimitrios Poulis, Evangelia Chaida, Theodoros Dimitroulas, Vasilios Kotsis, Eugenia Gkaliagkousi

**Affiliations:** 1 3rd Department of Internal Medicine, Papageorgiou Hospital, Aristotle University of Thessaloniki, Thessaloniki, Greece; 2 Fourth Department of Internal Medicine, Hippokration Hospital, Thessaloniki, Greece; 3 Second Medical Department, Hippokration Hospital, Thessaloniki, Greece

**Keywords:** Arterial stiffness, Myocardial perfusion, Potassium intake, Rheumatoid arthritis, Sodium intake, Urinary sodium-to-potassium ratio

## Abstract

**Introduction/objectives:**

Patients with rheumatoid arthritis (RA) are at increased cardiovascular risk. Rather than either sodium or potassium intake alone, the ratio of urinary sodium-to-potassium excretion has been introduced as a simple and useful indicator of diet quality and a more reliable index of cardiovascular risk assessment. We assessed the clinical impact of sodium-to-potassium ratio as a marker of cardiovascular health in patients with RA.

**Method:**

Sodium and potassium intake were assessed from 24-h urine samples, and urinary sodium-to-potassium ratio was calculated in patients with RA. Myocardial perfusion was assessed by measurement of subendocardial viability ratio (SEVR) using applanation tonometry. Pulse wave velocity and augmentation index were assessed as markers of arterial stiffness with the same device.

**Results:**

Among 61 patients with RA, only two presented an optimal sodium-to-potassium ratio of ≤ 1. In univariate analysis, urinary sodium excretion was significantly associated with high-density lipoprotein cholesterol (HDL-c) and uric acid. Potassium excretion positively correlated with estimated glomerular filtration rate (eGFR) and negatively with disease activity and inflammatory load. No associations were observed between markers of arterial stiffness and either urinary sodium excretion, potassium excretion, or their ratio. By contrast, both urinary sodium and urinary sodium-to-potassium ratio inversely correlated with SEVR, and these associations remained significant even after adjustment for other variables (beta =  − 0.247, *p* = 0.034, and beta =  − 0.247, *p* = 0.026, respectively).

**Conclusions:**

Findings from the present study suggest that in concordance with population-based studies, urinary sodium-to-potassium ratio might serve as an indicator of myocardial health in patients with autoimmune inflammatory diseases such as RA.

**Table:** 

**Key Points** • *Increased dietary sodium intake, decreased dietary potassium intake, and increased sodium-to-potassium ratio have been associated with adverse cardiovascular outcomes in longitudinal population-based cohorts*. • *In a population of high cardiovascular risk patients with RA, increased dietary sodium intake and increased urinary sodium-to-potassium ratio were both associated with impaired coronary microvascular perfusion*. • *Dietary potassium intake inversely correlated with disease activity and inflammatory load*. • *In patients with chronic inflammatory arthritis, interventions aiming at dietary modifications of sodium and potassium intake might positively affect both cardiovascular outcomes and disease-related parameters*.

## Introduction

Rheumatoid arthritis (RA) is an autoimmune disease with an estimated prevalence of around 1% in the general population, characterized by a predominantly female predisposition and chronic inflammatory load [1]. In patients with RA, cardiovascular events are a common extra-articular manifestation, and the cardiovascular risk associated with disease exceeds that of the general population by as much as 50% [2]. Over the past decade, advances marked in the field of RA treatment have focused on effective disease control, expanding the life expectancy and facilitating the life quality of these patients. Consequently, cardiovascular disease constitutes to date a major factor increasing mortality and compromising prognosis of RA patients [3]. Accelerated atherosclerosis in RA has been attributed inter alia to chronically active inflammation, antirheumatic drug interactions, and increased prevalence of cardiovascular risk factors, including hypertension, hypercholesterolemia, metabolic syndrome, and sedentary lifestyle [3, 4]. By contrast, the nutrient-specific behavior of patients with RA remains underinvestigated, especially in regard to cardiovascular and disease-related outcomes.

Diet constitutes a modifiable lifestyle factor with major implications for cardiovascular health. The impact of dietary components on vascular aging has been demonstrated in longitudinal cohorts, further supporting the notion that nutrient-specific behavior changes may mitigate cardiovascular outcomes throughout a lifetime [5]. In particular, a firm relationship of dietary sodium intake with cardiovascular risk has been established. Both the onset of hypertension and its fatal cardiovascular complications have been associated with increased sodium consumption among the general population, with an excessive number of deaths attributed to sodium overconsumption [6, 7]. However, rather than sodium excretion per se, the ratio between sodium and potassium has been recently acknowledged as a potent mediator of cardiovascular disease. Analysis from prospective cohorts has shown a dose–response association of higher sodium and lower potassium intakes, as measured in 24-h urine samples, with future cardiovascular risk [8, 9].

By contrast, there is a scarcity of studies examining the association between sodium and potassium consumption and markers of cardiovascular health in RA. We therefore investigated whether sodium and potassium intake and the ratio between urinary sodium and potassium excretion correlate with markers of cardiovascular disease (myocardial perfusion, vascular stiffness) in patients with RA and further explored potential associations with inflammation and disease-related parameters.

## Methods

This cross-sectional study recruited individuals ≥ 18 years attending the Rheumatology Outpatient Department of Hippokration Hospital, Thessaloniki, Greece, with an established diagnosis of RA according to the 1987 American College of Rheumatology criteria [10]. Exclusion criteria included recent cardiovascular events (myocardial infarction, unstable angina, stroke) within the past 6 months; estimated glomerular filtration rate ≤ 45 mL/min/m^2^; stage III-IV New York Heart Association (NYHA) heart failure; moderate-to-severe hepatic dysfunction; and presence of concomitant active malignancy, infection, or any disease with poor prognosis. The protocol was approved by the Aristotle University of Thessaloniki Ethics Committee (approval number: 377/27072017, approval date: July 27, 2017). The study was performed in accordance with the Helsinki Declaration, and all participants gave written informed consent before inclusion in the study.

Epidemiological data, medical history, and current medication use were recorded. All participants had their blood pressure recorded in the seated position according to current recommendations [11]. Hypercholesterolemia was defined according to current guidelines for the diagnosis and treatment of dyslipidemias that recommend consideration of patients with autoimmune or inflammatory disease as at least high-risk (low-density lipoprotein cholesterol (LDL-c) < 116 mg/dL) [12]. Hypertension was defined as office systolic and/or diastolic blood pressure ≥ 140/90 mmHg and/or current antihypertensive medication use [11]. Body mass index was calculated in kilogram per square meter. Blood samples were drawn after overnight fasting for routine biochemical measurements, including inflammatory markers (C-reactive protein (CRP), erythrocyte sedimentation rate (ESR)). Estimated glomerular filtration rate was calculated according to the Cockcroft-Gault equation. The 28-joint Disease Activity Score (DAS28) was calculated from the number of tender and swollen joints, the subjective estimate (0–100 scale) of pain/discomfort due to RA, and ESR values [13]. DAS28-ESR thresholds of ≤ 3.2, 3.2–5.1, and > 5.1 indicate low, moderate, and high disease activity, respectively. All vascular measurements were performed in the same temperature-controlled room, with the patient lying in the supine position for > 15 min. Participants were instructed to abstain from smoking, caffeine, tea or alcohol intake, and intense exercise for at least 2 h before assessments.

### Assessment of coronary microvascular perfusion

Subendocardial viability ratio (SEVR), also known as the Buckberg index, corresponds to the ratio of subendocardial to subepicardial blood flow and reflects the balance between myocardial oxygen supply and oxygen demand [14]. SEVR is a useful tool for evaluation of coronary microvascular perfusion as shown in various populations including RA [15]. SEVR was measured noninvasively with applanation tonometry in the radial artery (SphygmoCor device, AtCor Medical, Sydney, New South Wales, Australia). The ratio of the area under the diastolic segment to the area under the systolic segment of the derived aortic pressure waveform (diastolic pressure time index to tension time index) was automatically calculated to measure SEVR, with lower values, especially below 100%, indicating poorer perfusion of the subendocardium.

### Assessment of vascular stiffness

Arterial stiffness was evaluated by measurement of pulse wave velocity (PWV) and augmentation index (AIx) normalized to 75 bpm, using applanation tonometry with the same device. Carotid-femoral PWV, the gold-standard measure of arterial stiffness, was calculated according to a standard protocol as the distance traveled by the pulse wave between the carotid and the femoral sampling site, divided by time. The pulse wave was sequentially recorded in the carotid and femoral artery, and the distance traveled by the pulse wave was calculated from the difference between the distances from the sternal notch to the recording sites. An electrocardiogram was simultaneously recorded to calculate wave transit time. The abnormality threshold for PWV was 10 m/s according to international guidelines [11].

For the assessment of AIx, a micromanometer-tipped probe was used to detect the radial pulse waveform using the same device, and the aortic pressure waveform was analyzed from the radial pressure waveform. AIx was automatically calculated and corrected for the mean heart rate of 75 bpm, to exclude the influence of heart rate on the AIx.

### Assessment of urinary sodium and potassium excretion

Urinary sodium and potassium excretion was estimated from 24-h urine samples according to a standard protocol. Participants were advised to avoid any change in their usual dietary habits. The starting time of the urine collection was the timepoint in the morning after participants woke up and emptied their bladder for the first time; all urine for the remainder 24-h period was saved, stored, and kept cold in a urine container. The urine container was returned to the lab immediately after the 24-h urine collection was completed. Completeness of 24-h urine collections was determined based on urine volume and 24-h creatinine excretion [16, 17]. To avoid measurement errors associated with either undercollection or overcollection of 24-h urine samples, we excluded samples with urine volume < 500 mL or > 5000 mL and samples with reported duration of urine collection < 22 h or > 26 h, or estimated volume loss of > 100 mL. Urine sodium and potassium concentrations were estimated in mmol/24 h. Daily sodium intake was calculated with the following conversions: 93% of sodium is excreted into the urine, and 1 mmol of sodium is equivalent to 58.5 mg of NaCl [18]. Daily potassium excretion was calculated assuming a gastrointestinal absorption of 77%, and that 1 mmol potassium equaled 39 mg [19, 20].

### Statistical analysis

Data analysis was performed using Statistical Package for Social Sciences, SPSS Inc., Chicago, IL, USA, software, version 22. Results were expressed as frequencies for qualitative variables and as mean ± standard deviation (m ± SD) or median (interquartile range) for continuous variables. Pearson chi-square test was applied for comparison of frequencies. Differences between mean values were compared with Student’s *t*-test or Mann–Whitney test. Univariate correlations were assessed using the parametric Pearson or the nonparametric Spearman’s Rho correlation coefficient. Multiple linear regression analysis with the “Enter” method was used to identify the statistically significant predicting factors of SEVR. A probability value of *p* ≤ 0.05 was considered statistically significant.

## Results

A total of 61 patients with RA were included, with a mean age of 62.4 ± 9.9 years. Baseline characteristics of the study population are presented in Table 1. Routine hematological parameters, inflammatory markers, metabolic profile, and renal function, as well as urinary sodium and potassium measurements, are presented in Table 2. Consistent with the female predominance of the RA, the majority of patients were females. Patients suffered from long-standing disease, and the mean DAS28 score was indicative of low-to-moderate disease activity. The study population presented with cardiovascular risk factors and comorbidities. Approximately two-thirds (65%) of patients reported a history of hypertension. Measurement of lipid levels classified all patients as suffering from hypercholesterolemia (Table 2, part a). However, only 40.7% were aware of dyslipidemia, and only 23.3% were treated with statins (Table 1).

**Table 1:** Baseline characteristics of the study population

Variable	Patients with RA (*n* = 61)
Age (years)	62.4 ± 9.9
Female sex (%)	78.7
Disease duration (years)	10.4 ± 8.6
Systolic blood pressure (mmHg)	126.1 ± 16.7
Diastolic blood pressure (mmHg)	74.1 ± 8.3
Heart rate (bpm)	68 (15)
BMI (kg/m^2^)	27.1 ± 5.1
Obesity (%)	24.6
Overweight (%)	37.7
Normal weight (%)	36.0
Underweight (%)	1.6
Current smoking (%)	18.6
History of hypertension (%)	65.0
History of dyslipidemia (%)	40.7
Diabetes mellitus (%)	9.8
Coronary artery disease (%)	11.5
History of stroke (%)	4.9
DAS28	3.2 ± 1.1
Antirheumatic medication	
Methotrexate (%)	46.7
Corticosteroids (%)	35.0
Biologics (%)	40.0
Cardiovascular drugs	
RAAS inhibitors (%)	46.7
Calcium channel blockers (%)	25.0
Beta blockers (%)	30.0
Diuretics (%)	30.0
Antiplatelets (%)	11.7
Statins (%)	23.3

*RA* rheumatoid arthritis, *BMI* body mass index, *DAS28* disease activity score in 28 joints, *RAAS* renin–angiotensin–aldosterone system

**Table 2:** Laboratory and vascular measurements in the study population

	Patients with RA (*n* = 61)
*a. Routine laboratory measurements and inflammatory markers*	
ESR (mm/h)	18.0 (18.0)
CRP (mg/L)	3.2 (2.4)
WBC 10^3^/µL	6.5 (2.0)
Hematocrit (%)	37.6 ± 3.5
Hemoglobin (g/dL)	12.5 ± 1.3
Platelets (10^3^/µL)	220.9 ± 60.2
Glucose (mg/dL)	94 (18)
Urea (mg/dL)	36.2 ± 10.9
Creatinine (mg/dL)	0.85 (0.22)
eGFR (mL/min/m^2^)	79.2 ± 21.3
Uric acid (mg/dL)	4.5 ± 1.6
Glucose (mg/dL)	94.0 (18)
HDL-C (mg/dL)	53.0 (20.0)
LDL (mg/dL)	125.6 ± 32.5
Total cholesterol (mg/dL)	201.5 (38.5)
Triglycerides (mg/dL)	114.0 (55.0)
*b. Sodium and potassium excreted in the urine and estimated dietary intake*	
Urinary sodium (mmol)	107.1 ± 50.9
Urinary potassium (mmol)	42.5 (21.7)
Urinary sodium-to-potassium ratio	2.4 ± 1.0
Dietary salt intake (mg)	6737 ± 3212
Dietary potassium intake (mg)	2152 (1099)
*c. Vascular measurements in the study population*	
SEVR (%)	142.2 ± 23.3
PWV (m/s)	8.5 ± 2.3
AIx (%)	28.9 ± 6.6

*ESR* erythrocyte sedimentation rate, *CRP* C-reactive protein, *WBC* white blood cells, *eGFR* estimated glomerular filtration rate, *HDL-C* high-density lipoprotein cholesterol, *LDL-C* low-density lipoprotein cholesterol, *SEVR* subendocardial viability ratio, *PWV* pulse wave velocity, *AIx* augmentation index

Continuous variables are presented as mean ± SD or median (interquartile range) according to normality tests

As presented in Table 2, part b, 24-h urinary sodium and potassium excretion corresponds to daily salt intake of 6.7 ± 3.2 g, and daily potassium intake of 2.1 (1.1) g. Urinary sodium-to-potassium ratio was 2.4 ± 1.0; only two patients presented an optimal sodium-to-potassium ratio of ≤ 1.

### Association of sodium and potassium excretion with laboratory tests and cardiovascular risk factors

Of laboratory markers, urinary sodium excretion was significantly associated with HDL-c (*r* =  − 0.309, *p* = 0.016) and uric acid (*r* = 0.301, *p* = 0.028), while urinary potassium excretion was only associated with eGFR (*r* = 0.371, *p* = 0.004). Univariate analysis of urinary sodium-to-potassium ratio revealed significant associations with HDL-c (*r* =  − 0.316, *p* = 0.014), uric acid (*r* = 0.381, *p* = 0.005), and eGFR (*r* =  − 0.269, *p* = 0.039).

Urinary sodium, potassium, and their ratio correlated non-significantly with traditional cardiovascular risk factors (blood pressure, heart rate, body mass index, smoking, hypertension, diabetes, or dyslipidemia), as presented in detail in Table 3. In addition, urinary sodium, potassium, and their ratio did not significantly differ in subgroup analysis according to the use of diuretics or corticosteroids.

**Table 3:** Association of urinary sodium, potassium, and their ratio with traditional cardiovascular risk factors and comorbidities: (a) univariate correlation analysis, (b) subgroup analysis

	Urinary sodium excretion (mmol)	Urinary potassium excretion (mmol)	Urinary sodium-to-potassium ratio
a. Correlation analysis with cardiovascular risk factors			
	***r*/*P* value**	***r*/P value**	***r*/*P* value**
Age (years)	0.076/0.743	− 0.425/0.055	0.321/0.155
Systolic blood pressure (mmHg)	0.107/0.412	− 0.560/0.666	0.106/0.417
Diastolic blood pressure (mmHg)	0.022/0.868	0.015/0.906	0.009/0.948
Heart rate (bpm)	0.091/0.484	− 0.041/0.753	0.136/0.297
Body mass index (kg/m^2^)	0.056/0.669	0.024/0.852	− 0.024/0.854
b. Subgroup analysis according to cardiovascular risk factors and comorbidities			
	**Yes vs no (*P* value)**	**Yes vs no (*P* value)**	**Yes vs no (*P* value)**
Smoking	108.1 ± 40.6 vs 108.2 ± 53.6 (0.992)	43.0 (24.9) vs 41.3 (21.2) (0.397)	2.39 ± 1.33 vs 2.46 ± 0.94 (0.843)
Hypertension	106.7 ± 52.7 vs 108.2 ± 49.2 (0.917)	41.3 (18.9) vs 51.0 (27.4) (0.105)	2.54 ± 1.0 vs 2.25 ± 0.99 (0.299)
Diabetes	88.1 (78.4) vs 96.0 (75.2) (0.447)	32.3 (26.1) vs 43.2 (20.8) (0.058)	2.87 ± 1.04 vs 2.40 ± 0.99 (0.282)
Dyslipidemia	98.3 ± 40.1 vs 110.0 ± 55.2 (0.34)	41.2 (17.5) vs 42.5 (26.0) (0.478)	2.48 ± 0.99 vs 2.4 ± 1.0 (0.779)
Established cardiovascular disease	102.1 ± 52.7 vs 107.9 ± 51.6 (0.767)	41.3 (18.9) vs 42.8 (22.1) (0.695)	2.59 ± 1.23 vs 2.43 ± 0.98 (0.670)

Continuous variables are presented as mean ± SD or median (interquartile range) according to normality tests. Established cardiovascular disease refers to a history of coronary artery disease and/or stroke > 6 months prior to inclusion in the study

### Association of sodium and potassium excretion with disease-related parameters

In our study population, urinary potassium excretion was inversely associated with systemic inflammation, as indicated by ESR (*r* =  − 0.269, *p* = 0.039), and disease activity, as indicated by DAS28 (*r* =  − 0.387, *p* = 0.003). By contrast, neither urinary sodium excretion nor urinary sodium-to-potassium ratio was significantly associated with disease-related parameters, including inflammatory markers (ESR, CRP), disease duration, and disease activity.

### Association of sodium and potassium excretion with vascular measurements

Vascular measurements of coronary microvascular perfusion and large artery stiffening are presented in Table 2, part c. Two patients with RA (3.3%) exhibited SEVR values < 100%, and 15 patients (24.6%) presented PWV values ≥ 10 m/s. Neither PWV nor AIx was associated with urinary sodium, urinary potassium excretion, or their ratio.

By contrast, SEVR inversely correlated with both urinary sodium (*r* =  − 0.290, *p* = 0.027) and urinary sodium-to-potassium ratio (*r* =  − 0.270, *p* = 0.041), yet not with urinary potassium (*r* =  − 0.014, *p* = 0.914). Multivariate analysis for SEVR adjusting for cardiovascular risk factors revealed an independent association between both urinary sodium excretion (*r* =  − 0.247, *p* = 0.034) and urinary sodium-to-potassium ratio (*r* =  − 0.247, *p* = 0.026) (Table 4).

**Table 4:** Crude and corrected regression models for myocardial perfusion in the RA cohort

	**(a)** Univariate associations with SEVR		**(b)** Dependent variable: SEVR, *p* < 0.001, *R* square = 0.460, adjusted *R* square = 0.381						**(c)** Dependent variable: SEVR, *p* < 0.001, *R* square = 0.465, adjusted *R* square = 0.387					
Variable	***r***	***P***	**Unstand. C**		**Stand. C**	**95% CI for B**		***P***	**Unstand. C**		**Stand. C**	**95% CI for B**		**P**
			B	SD	Beta	LB	UB		B	SD	Beta	LB	UB	
Age (y)	0.180	0.177	0.271	0.317	0.115	− 0.367	0.908	0.398	0.509	0.309	0.217	− 0.112	1.131	0.106
Gender	− 0.163	0.221	− 13.849	6.580	− 0.249	− 27.078	− 0.620	0.041	− 11.587	6.394	− 0.208	− 24.442	1.268	0.076
Systolic blood pressure (mmHg)	− 0.252	0.056	− 0.338	0.183	− 0.242	− 0.706	0.029	0.070	− 0.410	0.178	− 0.293	− 0.768	− 0.052	0.026
Heart rate (bpm)	− 0.625	< 0.001	− 0.804	0.218	− 0.423	− 1.243	− 0.366	0.001	− 0.762	0.218	− 0.401	− 1.200	− 0.324	0.001
CRP (mg/L)	− 0.057	0.675	0.324	0.390	0.093	− 0.459	1.107	0.410	0.324	0.388	0.093	− 0.456	1.103	0.408
Total cholesterol (mg/dL)	0.070	0.606	0.100	0.073	0.149	− 0.048	0.247	0.182	0.086	0.073	0.128	− 0.061	0.232	0.246
Urinary sodium excretion (mmoL)	− 0.290	0.027	− 0.116	0.053	− 0.247	− 0.223	− 0.009	0.034						
Urinary sodium-to-potassium ratio	− 0.270	0.041							− 5.674	2.477	− 0.247	− 10.655	− 0.693	0.026

*RA* rheumatoid arthritis, *SEVR* subendocardial viability ratio, *CRP* C-reactive protein, *Unstand. C.* unstandardized coefficient*, Stand. C.* standardized coefficient, *CI* confidence intervals, *LB* lower bound, *UB* upper bound

## Discussion

In the present study, we showed for the first time that urinary sodium-to-potassium ratio correlates with microvascular myocardial perfusion in a population of high cardiovascular risk patients with chronic inflammatory disease. By contrast, markers of large artery stiffening were not associated with urinary sodium excretion, urinary potassium excretion, or their ratio. These findings add new evidence to the growing body of evidence regarding the importance of dietary sodium and potassium intake in terms of cardiovascular health, in a well-defined population of patients with RA. In combination with the herein observed associations of potassium intake with disease-related parameters, clinical implications of the study findings extend to adopting dietary patterns for patients with RA aiming at increasing potassium intake and decreasing sodium intake. Whether such strategies would ameliorate disease progression and improve cardiovascular outcomes in RA warrants further investigation.

Urinary sodium-to-potassium ratio has been acknowledged as a potent biomarker of cardiovascular diseases [9]. Restriction of sodium intake and augmentation of potassium intake is unanimously advocated from current international guidelines in the field of cardiovascular medicine towards cardiovascular risk reduction [21]. Adopting such dietary patterns could emerge as particularly important in chronic inflammatory diseases including RA, which is associated with substantial residual cardiovascular risk. Of note, cardiovascular risk in RA cannot be presently accurately quantified by use of general or disease-adapted risk calculators [3]. Hence, it is imperative to introduce novel biomarkers with proven cost-effectiveness and wide-scale applicability towards more accurate cardiovascular risk stratification in patients with RA. Findings from the present study showing an association between urinary-to-potassium ratio and myocardial microvascular perfusion further reinforce this concept, although validation from appropriately designed studies is warranted.

By contrast, urinary sodium and urinary sodium-to-potassium ratio were not associated with markers of large artery stiffening in the present population of patients with chronic autoimmune inflammation and cardiovascular risk factors. This finding implies that large artery stiffening may be dependent predominantly on other factors rather than sodium intake, as previously documented in other, more well-defined populations [22]. On the other hand, the effects of increased sodium consumption and disturbed balance between sodium and potassium intake on the heart have been well documented. Such dietary patterns are widely known to promote fluid retention, vascular wall remodeling, and endothelial damage, leading to vascular injury and, subsequently, cardiovascular morbidity and mortality. Potential underlying mechanisms involve activation of the sympathetic nervous system, insulin resistance, and the renin–angiotensin–aldosterone system [23].

In addition to its role as a cardiovascular risk indicator, a potential role of increased sodium load in the pathogenesis of chronic inflammatory conditions has been proposed. In tandem with genetics, several environmental factors have been associated with RA predisposition, including dietary patterns [24, 25]. Increased dietary sodium consumption, common in Western countries, has been associated with an increased risk of RA [26]. Recently, a correlation was made between sodium intake and the emergence of autoimmune conditions such as RA, multiple sclerosis, and Crohn’s disease [27]. High levels of sodium consumption exacerbate inflammation in experimental models of autoimmunity, whereas low levels seem to contain inflammation [28, 29]. In our study, only increased dietary potassium intake was associated with lower levels of systemic inflammation and suppressed disease activity. Consistent with our findings, high potassium intake, especially in the context of hypokalemia, has been previously correlated with lower rates of reported arthralgias [30] and overall enhanced disease control [31]. Whether dietary patterns aiming at increasing potassium intake and decreasing sodium intake would be beneficial in terms of disease activity warrants further investigation.

Few studies have previously addressed sodium and potassium intake in patients with RA, and none in regard to vascular biomarkers or surrogate markers of cardiovascular disease. In a previous study using the Kawasaki formula in spot urine samples, the estimated sodium-to-potassium excretion ratio was found to be substantially increased in the RA group compared to controls, although daily sodium excretion was found to be equally elevated in the RA group [32]. In the same study, the urinary sodium-to-potassium excretion ratio was not associated with the inflammatory burden of RA patients [32]. By contrast, analysis of a cohort database of 366 RA individuals showed that the urinary sodium-to-potassium ratio, also measured in spot urine, was associated with the prevalence of hypertension as well as disease activity as assessed with DAS28 [33]. In a nationwide study conducted on the Korean population, 24-h sodium excretion was elevated in RA patients compared to their counterparts, although the constricted nature of the sample size did not allow for an inspection of salt sensitivity in RA [34]. In a small case–control study of 20 patients with RA, sodium excretion was higher for patients with early RA compared to their matched controls. Patients with erosions at diagnosis had greater levels of sodium excretion than those with non-erosive disease, although sodium excretion was not directly related to disease activity [35].

Further implications from the present study can be summarized as follows. First, estimated daily salt consumption in the study population exceeded the daily amount unanimously advocated by current guidelines issued by the European Society of Cardiology/European Society of Hypertension and the World Health Organization (WHO) (salt restriction < 5 g, equivalent to approximately 2 g sodium, daily) [11, 36, 37]. At the same time, potassium consumption in the present cohort of RA patients was substantially lower than the recommended WHO amount of at least 3510 mg per day [38]. Only 3.3% of the study participants presented an optimal urinary sodium-to-potassium ratio of ≤ 1. Health care policies developed for patients suffering from chronic inflammatory diseases such as RA need to highlight the importance of adopting healthy dietary patterns, with special emphasis on dietary salt restriction and increased potassium consumption, preferably via dietary modification [39].

Second, traditional cardiovascular risk factors were inadequately controlled in the study population. This holds true especially for hypercholesterolemia. Impressively, although all patients in our cohort were classified as suffering from hypercholesterolemia according to current guidelines [12], only 40.7% were aware of dyslipidemia and less than one in four were prescribed statins. In addition, only 65% of the study participants were aware of hypertension. These findings are consistent with previously published data. The recent SURF-RA study from more than 14,500 patients with RA across three continents confirmed the increased prevalence and inadequate control of cardiovascular risk factors, with 62% of patients diagnosed as hypertensives and another 63% presenting with LDL-c of > 2.5 mmol/L [40]. Altogether, these findings call for a more careful evaluation of cardiovascular risk in patients with chronic inflammatory diseases and highlight the need to overcome therapeutic inertia resulting in suboptimal treatment and control of cardiovascular risk factors and comorbidities.

Third, neither urinary sodium nor potassium excretion was associated with blood pressure or presence of hypertension in our study population. Although dietary sodium and potassium intake are traditionally considered to predominantly affect blood pressure levels, this association is more complex in a population with chronic inflammatory load, and the absence of such an association cannot be merely attributed to the small study sample. Of note, the notion of salt sensitivity is involved in the correlation between sodium intake and hypertension, as blood pressure elevation is not contingent on sodium intake in all individuals [41]. Various classes of antihypertensive drugs were prescribed in our patients, including diuretics, which promote the excretion of sodium and potassium in the urine. In addition, around one-third of the study participants were receiving corticosteroids that promote a state of water and salt retention through activation of the glucocorticoid and mineralocorticoid receptors responsible for regulating salt and water homeostasis [42]. Although subgroup analysis for urinary sodium, potassium, and their ratio according to diuretics or corticosteroid use did not reveal significant differences, our study was not powered to allow for a stratified analysis according to the use of glucocorticosteroids and/or diuretics.

Strengths of our study include a solid methodological approach and the use of well-acknowledged vascular biomarkers. Sodium and potassium intake were assessed by means of 24-h urine collections, which represents the most reliable method for their dietary assessment [43]. Limitations of our study include the absence of a control group; this limits the generalizability of the findings and makes them only relevant to patients with rheumatoid arthritis. Still, even in the absence of a control group, it could be concluded that the estimated daily consumption of sodium and potassium was beyond optimal. The study is limited by its cross-sectional design, and future prospective studies are needed to verify these results. We recruited a real-life cohort of RA patients, who presented several cardiovascular risk factors and comorbidities and received antihypertensive and antirheumatic treatment. These may have acted as confounding factors that enhance or obscure potential associations with sodium and potassium intake. The small number of patients with a history of coronary artery disease or stroke does not allow for a stratified analysis. In addition, we are not able to provide reliable data regarding heart failure since patients with overt clinical signs and symptoms were excluded, and those included were not referred to a cardiologist for the purposes of our study. Therefore, the multimorbidity of the study population does not allow for concise conclusions regarding the net effect of RA on the observed associations since our study was underpowered to adjust for the presence of cardiovascular comorbidities. However, the included patient characteristics reflect those encountered in real-world clinical practice, and the present study findings should be interpreted in this prism.

In conclusion, in a real-life cohort of patients with RA, urinary sodium-to-potassium ratio may serve as an index of myocardial health. Increased dietary sodium intake inversely correlated with impaired coronary microvascular perfusion, whereas dietary potassium intake inversely correlated with disease activity and inflammatory load. In patients with chronic inflammatory diseases, interventions aiming at dietary modifications of sodium and potassium intake might positively affect both cardiovascular outcomes and disease-related parameters, but this hypothesis needs to be tested in appropriately designed studies.

## Data Availability

The datasets generated during and/or analyzed during the current study are available from the corresponding author on reasonable request.

## References

[1] Jahid M, Khan KU, Rehan-Ul-Haq null, Ahmed RS (2023) Overview of rheumatoid arthritis and scientific understanding of the disease. Mediterr J Rheumatol 34:284–291. 10.31138/mjr.20230801.oo 10.31138/mjr.20230801.oo PMC10628871 37941854

[2] Avina-Zubieta JA, Thomas J, Sadatsafavi M, Lehman AJ, Lacaille D (2012) Risk of incident cardiovascular events in patients with rheumatoid arthritis: a meta-analysis of observational studies. Ann Rheum Dis 71:1524–1529. 10.1136/annrheumdis-2011-200726 22425941 10.1136/annrheumdis-2011-200726

[3] Anyfanti P, Ainatzoglou A, Angeloudi E, Michailou O, Defteraiou K, Bekiari E, Kitas GD, Dimitroulas T (2024) Cardiovascular risk in rheumatoid arthritis: considerations on assessment and management. Mediterr J Rheumatol 35:402–410. 10.31138/mjr.310824.cri 39463875 10.31138/mjr.310824.cri PMC11500121

[4] Anyfanti P, Dara A, Angeloudi E, Bekiari E, Dimitroulas T, Kitas GD (2021) Monitoring and managing cardiovascular risk in immune mediated inflammatory diseases. J Inflamm Res 14:6893–6906. 10.2147/JIR.S276986 34934338 10.2147/JIR.S276986 PMC8684400

[5] Osborne M, Bernard A, Falkowski E, Peterson D, Vavilikolanu A, Komnenov D (2023) Longitudinal associations of dietary fructose, sodium, and potassium and psychological stress with vascular aging index and incident cardiovascular disease in the CARDIA cohort. Nutrients 16:127. 10.3390/nu16010127 38201956 10.3390/nu16010127 PMC10780647

[6] Mozaffarian D, Fahimi S, Singh GM et al (2014) Global sodium consumption and death from cardiovascular causes. N Engl J Med 371:624–634. 10.1056/NEJMoa1304127 25119608 10.1056/NEJMoa1304127

[7] Jiang L, Shen W, Wang A, Fang H, Wang Q, Li H, Liu S, Shen Y, Liu A (2024) Cardiovascular disease burden attributable to high sodium intake in China: a longitudinal study from 1990 to 2019. Nutrients 16:1307. 10.3390/nu16091307 38732554 10.3390/nu16091307 PMC11085757

[8] Wang DD, Li Y, Nguyen X-MT et al (2022) Dietary sodium and potassium intake and risk of non-fatal cardiovascular diseases: the Million Veteran Program. Nutrients 14:1121. 10.3390/nu14051121 35268096 10.3390/nu14051121 PMC8912456

[9] Ma Y, He FJ, Sun Q et al (2022) 24-hour urinary sodium and potassium excretion and cardiovascular risk. N Engl J Med 386:252–263. 10.1056/NEJMoa2109794 34767706 10.1056/NEJMoa2109794 PMC9153854

[10] Arnett FC, Edworthy SM, Bloch DA, McShane DJ, Fries JF, Cooper NS, Healey LA, Kaplan SR, Liang MH, Luthra HS (1988) The American Rheumatism Association 1987 revised criteria for the classification of rheumatoid arthritis. Arthritis Rheum 31:315–324. 10.1002/art.1780310302 3358796 10.1002/art.1780310302

[11] Mancia G, Kreutz R, Brunström M et al (2023) 2023 ESH guidelines for the management of arterial hypertension the Task Force for the management of arterial hypertension of the European Society of Hypertension: endorsed by the International Society of Hypertension (ISH) and the European Renal Association (ERA). J Hypertens 41:1874–2071. 10.1097/HJH.0000000000003480 37345492 10.1097/HJH.0000000000003480

[12] Katsiki N, Filippatos T, Vlachopoulos C et al (2024) Executive summary of the Hellenic Atherosclerosis Society guidelines for the diagnosis and treatment of dyslipidemias - 2023. Atherosclerosis Plus 55:74–92. 10.1016/j.athplu.2024.01.004 38425675 10.1016/j.athplu.2024.01.004 PMC10901915

[13] Prevoo ML, van ’t Hof MA, Kuper HH, van Leeuwen MA, van de Putte LB, van Riel PL (1995) Modified disease activity scores that include twenty-eight-joint counts. Development and validation in a prospective longitudinal study of patients with rheumatoid arthritis. Arthritis Rheum 38:44–48. 10.1002/art.1780380107 10.1002/art.1780380107 7818570

[14] Buckberg GD, Fixler DE, Archie JP, Hoffman JI (1972) Experimental subendocardial ischemia in dogs with normal coronary arteries. Circ Res 30:67–81. 10.1161/01.res.30.1.67 5007529 10.1161/01.res.30.1.67

[15] Xie H, Gao L, Fan F, Gong Y, Zhang Y (2024) Research progress and clinical value of subendocardial viability ratio. J Am Heart Assoc 13:e032614. 10.1161/JAHA.123.032614 38471822 10.1161/JAHA.123.032614 PMC11009993

[16] Ljungman S, Aurell M, Hartford M, Wikstrand J, Wilhelmsen L, Berglund G (1981) Sodium excretion and blood pressure. Hypertension 3:318–326. 10.1161/01.hyp.3.3.318 7251092 10.1161/01.hyp.3.3.318

[17] Schachter J, Harper PH, Radin ME, Caggiula AW, McDonald RH, Diven WF (1980) Comparison of sodium and potassium intake with excretion. Hypertension 2:695–699. 10.1161/01.hyp.2.5.695 7419270 10.1161/01.hyp.2.5.695

[18] Lucko AM, Doktorchik C, Woodward M et al (2018) Percentage of ingested sodium excreted in 24-hour urine collections: a systematic review and meta-analysis. J Clin Hypertens (Greenwich) 20:1220–1229. 10.1111/jch.13353 30101426 10.1111/jch.13353 PMC8030843

[19] Guideline: potassium intake for adults and children. https://www.who.int/publications/i/item/9789241504829. Accessed 11 Mar 2025 23617019

[20] Holbrook JT, Patterson KY, Bodner JE, Douglas LW, Veillon C, Kelsay JL, Mertz W, Smith JC (1984) Sodium and potassium intake and balance in adults consuming self-selected diets. Am J Clin Nutr 40:786–793. 10.1093/ajcn/40.4.786 6486085 10.1093/ajcn/40.4.786

[21] Jackson SL, Cogswell ME, Zhao L et al (2018) Association between urinary sodium and potassium excretion and blood pressure among adults in the United States: National health and nutrition examination survey, 2014. Circulation 137:237–246. 10.1161/CIRCULATIONAHA.117.029193 29021321 10.1161/CIRCULATIONAHA.117.029193 PMC5771856

[22] Triantafyllou A, Anyfanti P, Gkaliagkousi E, Zabulis X, Vamvakis A, Gkolias V et al (2018) Association of urinary sodium excretion with vascular damage: a local kidney effect, rather than a marker of generalized vascular impairment. Int J Hypertens 2018:7620563. 10.1155/2018/7620563 30643643 10.1155/2018/7620563 PMC6311280

[23] Grillo A, Salvi L, Coruzzi P, Salvi P, Parati G (2019) Sodium intake and hypertension. Nutrients 11:1970. 10.3390/nu11091970 31438636 10.3390/nu11091970 PMC6770596

[24] Malmström V, Catrina AI, Klareskog L (2017) The immunopathogenesis of seropositive rheumatoid arthritis: from triggering to targeting. Nat Rev Immunol 17:60–75. 10.1038/nri.2016.124 27916980 10.1038/nri.2016.124

[25] Sharif K, Amital H, Shoenfeld Y (2018) The role of dietary sodium in autoimmune diseases: the salty truth. Autoimmun Rev 17:1069–1073. 10.1016/j.autrev.2018.05.007 30213699 10.1016/j.autrev.2018.05.007

[26] Salgado E, Bes-Rastrollo M, de Irala J, Carmona L, Gómez-Reino JJ (2015) High sodium intake is associated with self-reported rheumatoid arthritis: a cross sectional and case control analysis within the SUN cohort. Medicine (Baltimore) 94:e0924. 10.1097/MD.0000000000000924 26376372 10.1097/MD.0000000000000924 PMC4635786

[27] Kleinewietfeld M, Manzel A, Titze J, Kvakan H, Yosef N, Linker RA, Muller DN, Hafler DA (2013) Sodium chloride drives autoimmune disease by the induction of pathogenic TH17 cells. Nature 496:518–522. 10.1038/nature11868 23467095 10.1038/nature11868 PMC3746493

[28] Wu C, Yosef N, Thalhamer T, Zhu C, Xiao S, Kishi Y, Regev A, Kuchroo VK (2013) Induction of pathogenic TH17 cells by inducible salt-sensing kinase SGK1. Nature 496:513–517. 10.1038/nature11984 23467085 10.1038/nature11984 PMC3637879

[29] Sehnert B, Pohle S, Heuberger C, Rzepka R, Seidl M, Nimmerjahn F, Chevalier N, Titze J, Voll RE (2021) Low-salt diet attenuates B-cell- and myeloid-cell-driven experimental arthritides by affecting innate as well as adaptive immune mechanisms. Front Immunol 12:765741. 10.31138/mjr.32.1.3 34925335 10.3389/fimmu.2021.765741 PMC8678127

[30] Rastmanesh R, Abargouei AS, Shadman Z, Ebrahimi AA, Weber CE (2008) A pilot study of potassium supplementation in the treatment of hypokalemic patients with rheumatoid arthritis: a randomized, double-blinded, placebo-controlled trial. J Pain 9:722–731. 10.1016/j.jpain.2008.03.006 18468955 10.1016/j.jpain.2008.03.006

[31] Murakami I, Murakami K, Hashimoto M et al (2020) Intake frequency of vegetables or seafoods negatively correlates with disease activity of rheumatoid arthritis. PLoS One 15:e0228852. 10.1371/journal.pone.0228852 32053642 10.1371/journal.pone.0228852 PMC7018088

[32] Carranza-Leon D, Octaria R, Ormseth MJ, Oeser A, Solus JF, Zhang Y, Okafor CR, Titze J, Michael Stein C, Chung CP (2018) Association between urinary sodium and potassium excretion and blood pressure and inflammation in patients with rheumatoid arthritis. Clin Rheumatol 37:895–900. 10.1007/s10067-017-3935-8 29243056 10.1007/s10067-017-3935-8 PMC5912908

[33] Minamino H, Katsushima M, Hashimoto M et al (2021) Urinary sodium-to-potassium ratio associates with hypertension and current disease activity in patients with rheumatoid arthritis: a cross-sectional study. Arthritis Res Ther 23:96. 10.1186/s13075-021-02479-x 33773587 10.1186/s13075-021-02479-x PMC8004419

[34] Bae JH, Shin MY, Kang EH, Lee YJ, Ha YJ (2021) Association of rheumatoid arthritis and high sodium intake with major adverse cardiovascular events: a cross-sectional study from the seventh Korean National Health and Nutrition Examination Survey. BMJ Open 11:e056255. 10.1136/bmjopen-2021-056255 34930746 10.1136/bmjopen-2021-056255 PMC8689190

[35] Marouen S, du Cailar G, Audo R, Lukas C, Vial G, Tournadre A et al (2012) Sodium excretion is higher in patients with rheumatoid arthritis than in matched controls. PLoS ONE 12:e0186157. 10.1371/journal.pone.0186157 10.1371/journal.pone.0186157 PMC5640209 29028829

[36] Whelton PK, Appel LJ, Sacco RL et al (2012) Sodium, blood pressure, and cardiovascular disease: further evidence supporting the American Heart Association sodium reduction recommendations. Circulation 126:2880–2889. 10.1161/CIR.0b013e318279acbf 23124030 10.1161/CIR.0b013e318279acbf

[37] McEvoy JW, McCarthy CP, Bruno RM, Brouwers S, Canavan MD, Ceconi C et al (2024) 2024 ESC guidelines for the management of elevated blood pressure and hypertension. Eur Heart J 45:3912–4018. 10.1093/eurheartj/ehae178 39210715 10.1093/eurheartj/ehae178

[38] WHO issues new guidance on dietary salt and potassium. https://www.who.int/news/item/31-01-2013-who-issues-new-guidance-on-dietary-salt-and-potassium. Accessed 18 Mar 2025

[39] Gioia C, Lucchino B, Tarsitano MG, Iannuccelli C, Di Franco M (2020) Dietary habits and nutrition in rheumatoid arthritis: can diet influence disease development and clinical manifestations? Nutrients 12:1456. 10.3390/nu12051456 32443535 10.3390/nu12051456 PMC7284442

[40] Semb AG, Ikdahl E, Kerola AM et al (2022) A Clinical Audit of Cardiovascular Risk Factors and Disease in Patients with Rheumatoid Arthritis - SURF-RA. Mediterr J Rheumatol 33:201–217. 10.31138/mjr.33.2.201 36128215 10.31138/mjr.33.2.201 PMC9450194

[41] Weinberger MH (1996) Salt sensitivity of blood pressure in humans. Hypertension 27:481–490. 10.1161/01.hyp.27.3.481 8613190 10.1161/01.hyp.27.3.481

[42] Sholter DE, Armstrong PW (2000) Adverse effects of corticosteroids on the cardiovascular system. Can J Cardiol 16:505–511 10787466

[43] McLean RM (2014) Measuring population sodium intake: a review of methods. Nutrients 6:4651–4662. 10.3390/nu611465 25353661 10.3390/nu6114651 PMC4245554

